# Exploring cancer in *LRRK2* mutation carriers and idiopathic Parkinson's disease

**DOI:** 10.1002/brb3.858

**Published:** 2017-12-07

**Authors:** Bjørg Johanne Warø, Jan O. Aasly

**Affiliations:** ^1^ Department of Neuroscience Norwegian University of Science and Technology Trondheim Norway; ^2^ Department of Neurology St. Olav's Hospital Trondheim Norway

**Keywords:** cancer, genetics, LRRK2, Parkinson

## Abstract

**Objectives:**

To compare the risk of non‐skin cancer in *LRRK2* mutation carriers and individuals with idiopathic Parkinson's disease (iPD), explore the age at which *LRRK2* mutation carriers have cancer compared to iPD subjects, and clarify whether certain cancers are more closely associated with the *LRRK2* mutation than iPD.

**Materials and Methods:**

Demographic data and cancer outcomes from 830 iPD patients and 103 *LRRK2* mutation carriers (27 with PD) were retrospectively collected. Oncologic data were obtained from the Cancer Registry of Norway and included cancer type and age at cancer. All study participants were of Norwegian ethnicity.

**Results:**

*LRRK2* mutation carriers have increased risk of non‐skin cancer compared with iPD subjects (OR 2.09; 95% CI 1.16–3.77; *p *=* *.015). A significant association was found between the mutation and breast cancer in women (OR 4.58; 95% CI 1.45–14.51; *p *=* *.010). No other associations between harboring a *LRRK2* mutation and specific cancer types were uncovered.

**Conclusion:**

*LRRK2* mutation carriers have an increased risk of non‐skin cancer compared with iPD subjects, which was mainly driven by the association between harboring the mutation and breast cancer in women. The increased risk is likely independent of ethnicity.

## 1 INTRODUCTION

It has long been believed that patients with Parkinson's disease (PD) have a reduced risk of cancer. In the 1960s, Hoehn and Yahr examined the cause of death in 194 PD patients and found that 24 had died of cancer, while the expected number was 41 based on the New York population from which the patients came (Hoehn & Yahr, 1967). In the 1980s, Jansson and Jankovic examined 406 PD patients and found that all types of cancer, except thyroid cancer and malignant melanoma, occurred less frequently than in the normal population (Jansson & Jankovic, 1985). Since then evidence of a lower cancer risk among PD patients has been fairly consistent; a meta‐analysis of 29 studies including over 100,000 PD patients found an aggregated risk for non‐skin cancer in PD patients compared with controls of 0.73 (95% confidence interval [CI], 0.63–0.83; Bajaj, Driver, & Schernhammer, 2010). A large Danish population‐based cohort study found a lower frequency of most types of cancer, with the exception of melanoma, non‐melanoma skin cancer, and breast cancer, in PD patients compared with the general population (Rugbjerg, Friis, Lassen, Ritz, & Olsen, 2012).

At first glance, PD and cancer seem unlike; cancer is characterized by uncontrolled cell growth, while PD is caused by untimely cell death. It was previously assumed that PD was a sporadic disease with little or no genetic component. The reduced cancer frequency in PD could therefore be attributed to environmental or lifestyle factors, especially since the low prevalence of cancer in PD is most evident for cancer types associated with smoking (Bajaj et al., 2010; Rugbjerg et al., 2012). However, over the past two decades, genetic research has led to the new insight into the pathogenic mechanisms of PD. Dominant and recessive gene mutations, and risk loci have been described in both familial and sporadic PD (Klein & Westenberger, 2012). Several of these genes may regulate cell cycle, and some of the mutations have been implicated in cancer (Inzelberg & Jankovic, 2007; West, Dawson, & Dawson, 2005). This may therefore imply different cancer frequencies in genetic versus idiopathic PD. Genetic forms of PD constitute 3–5% of sporadic PD cases and 30% of familial (Klein & Westenberger, 2012). Approximately, 10% of PD patients report a family history of the disease (Klein & Westenberger, 2012), but most gene mutations are rare, making it difficult to assess the cancer frequency in the various forms of genetic PD. Mutations in the *LRRK2* gene cause autosomal dominant PD, and are the most common genetic cause of PD (Healy et al., 2008). Several studies have assessed the cancer frequency in *LRRK2*‐associated PD. The majority of which have focused on the *G2019S* mutation, but *R1441H* has also been studied. Although these studies have been inconclusive, the majority indicate an increased risk of cancer in PD patients with the *G2019S* mutation compared with idiopathic PD (iPD; Agalliu et al., 2015; Allegra, Tunesi, Cilia, Pezzoli, & Goldwurm, 2014; Inzelberg et al., 2012; Ruiz‐Martinez et al., 2014; Saunders‐Pullman et al., 2010). This holds particular true for breast cancer in women (Agalliu et al., 2015; Inzelberg et al., 2012).

The objectives of this study were to compare the risk of non‐skin cancer in *LRRK2* mutation carriers and individuals with iPD, explore the age at which *LRRK2* mutation carriers have cancer compared to iPD subjects, and clarify whether certain cancers are more closely associated with the *LRRK2* mutation than iPD.

## 2 MATERIALS AND METHODS

Consecutive PD patients have been followed longitudinally since 1998 by one movement disorder specialist (JOA) at three outpatient clinics in central Norway, including St. Olav's Hospital (Trondheim University Hospital). All patients were asked to invite first‐degree relatives to participate in research. Peripheral blood was screened for several pathogenic PD mutations as previously described (Aasly et al., 2005, 2010; Johansen, Hasselberg, White, Farrer, & Aasly, 2010). Once LRRK2‐associated PD was identified in 2004, first‐ and second‐degree relatives of LRRK2‐PD patients were invited to partake in research.

By December 31st 2013, the iPD cohort consisted of 830 individuals, whereas the LRRK2 cohort consisted of 103 individuals: 27 manifesting *LRRK2* mutation carriers (LRRK2 PD+) and 76 non‐manifesting *LRRK2* mutation carriers (LRRK2 PD−). All the *LRRK2* mutation carriers harbored the *G2019S* mutation except 9 who were *N1437H* carriers. Two of these had PD, while seven were non‐manifesting carriers. All subjects of the iPD and LRRK2 cohorts were of Norwegian ethnicity.

All PD patients met the inclusion criteria for PD by the UK Parkinson's Disease Society Brain Bank Clinical Diagnostic Criteria (Hughes, Daniel, Kilford, & Lees, 1992).

The study was approved by the Institutional Review Board, and all study participants gave written informed consent.

Demographic data and cancer outcomes from 830 iPD patients and 103 *LRRK2* mutation carriers (27 with PD) were retrospectively collected. Oncologic data were obtained from the Cancer Registry of Norway (CRN). The Cancer Registry of Norway was established in 1951. The aim of the registry is to conduct population‐based cancer research by identifying all cancer cases in Norway. All medical doctors in the country are instructed by law to notify new cancer cases to the registry. The following must be reported to the CRN: all malignant neoplasms and precancerous disorders, and all benign tumors of the central nervous system and the meninges. Cancer notifications include clinical and pathological information, and are combined with the unique personal identification number used in Norway. The oncologic data obtained in this study included cancer type and age at cancer. Type of cancer was coded according to ICD‐10.

All statistical analyses were conducted using IBM Statistical package for the Social Sciences version 24 (SPSS Inc., Chicago, IL, USA) for Mac OS X.

Demographic, disease characteristics, and cancer outcomes were compared using student t‐tests (for continuous, normally distributed variables) and chi‐squared or Fisher's Exact tests (for categorical variables).

Logistic regression models were used to examine the associations between all non‐skin cancers combined (excluding melanoma and non‐melanoma skin cancer) and various cancer types and the *LRRK2*‐mutation with age and sex as explanatory variables.

The Kaplan–Maier method was used to estimate survival curves for age at cancer diagnosis for both *LRRK2*‐mutation carriers and iPD‐patients censoring at age at end of study or age at death. The log‐rank test was applied to compare the survival curves.

## 3 RESULTS

Demographic, clinical, and general cancer characteristics are presented in Table 1, while cancer types are presented in Table 2. In all, 27 PD patients, corresponding to 3.1% of the total PD population in central Norway, carried a *LRRK2* mutation (Johansen et al., 2010). As the LRRK2 cohort consisted of both manifesting (LRRK2 PD+) and non‐manifesting carriers (LRRK2 PD−), it was significantly younger than the iPD cohort. It was also more likely to include women (50.5 vs. 37.2%, *p *=* *.009). Age at PD onset and disease duration was similar in both groups. A total of 144 incidents of non‐skin cancers were found in both cohorts. Non‐skin cancers were diagnosed in 18 (17.5%) in the LRRK2 group and 126 (15.1%) in the iPD group. The proportion of non‐skin cancers was slightly higher in the LRRK2 PD+ compared with the iPD subjects, 22.2 versus 15.1%, respectively. No incidents of lung, bladder, kidney, or thyroid cancers were reported in the LRRK2 group. The mean age at non‐skin cancer was younger in the LRRK2 subjects (60.5 years) than the iPD subjects (66.3 years). This difference tended toward significance, *p *=* *.058. Six of 27 LRRK2 PD+ individuals were diagnosed with cancer. Four (67%) of these were diagnosed with cancer before PD and two (33%) after PD. In all, 12 LRRK2 PD− subjects had cancer. Among the iPD subjects with cancer, 45 (33%) were diagnosed with cancer before PD, while 92 (67%) were diagnosed with cancer after PD.

**Table 1:** Demographic and clinical features for *LRRK2* mutation carriers and iPD subjects

	LRRK2 (*n* = 103)	iPD (*n* = 830)	*p*‐value
Sex			
Male, *n* (%)	51 (49.5)	521 (62.8)	.009
Female, *n* (%)	52 (50.5)	309 (37.2)	
Age, years mean ± *SD* (range)	60.4 ± 16.8 (27.5, 95.5)	72.5 ± 10.0 (27.7, 96.8)	<.001
Age at PD onset, years mean ± *SD* (range)	60.3 ± 11.9 (37, 80)	59.4 ± 10.8 (22, 87)	.682
PD duration, years mean ± *SD* (range)	11.8 ± 8.8 (2.4, 29.5)	12.2 ± 6.7 (1.4, 46.9)	.833
Non‐skin cancer *n* (%)	18 (17.5)	126 (15.1)	.521
Age at non‐skin cancer, years mean ± *SD* (range)	60.5 ± 14.2 (33.3, 79.7)	66.3 ± 11.8 (19.6, 88.6)	.058

**Table 2:** Cancer types in *LRRK2* mutation carriers and in iPD subjects

Cancer type	Total *n* = 933, *n* (%)	LRRK2 *n* = 103, *n* (%)	iPD *n* = 830, *n* (%)
Colorectal	27 (2.9)	2 (1.9)	25 (3.0)
Lung	5 (0.5)	0	5 (0.6)
Breast – women^a^	16 (4.4)^a^	5 (9.6)^a^	11 (3.6)^a^
Prostate – men^a^	37 (6.5)^a^	3 (5.9)^a^	34 (6.5)^a^
Kidney	3 (0.3)	0	3 (0.4)
Bladder	8 (0.9)	0	8 (1.0)
Thyroid	2 (0.2)	0	2 (0.2)
Lymphoma/Hematologic	14 (1.5)	2 (1.9)	12 (1.4)
Meningioma	10 (1.1)	2 (1.9)	8 (1.0)
Other	22 (2.4)	4 (3.9)	18 (2.2)

a Percentages of sex specific cancers are based on the number of men and women.

Table 3 provides associations of *LRRK2* mutation with all non‐skin cancers and various cancer outcomes using logistic regression models adjusting for age and sex. There was a significant association between harboring a *LRRK2* mutation all non‐skin cancers combined (OR 2.09; 95% CI 1.16–3.77; *p *=* *.015). A significant association between the *LRRK2* mutation and breast cancer in women adjusted was also seen (OR 4.58; 95% CI 1.45–14.51; *p *=* *.010). After removing the breast cancer cases, a non‐significant association between harboring a *LRRK2* mutation and cancer when adjusting for age and sex was found (OR 1.73; 95% CI 0.90–3.36; *p *=* *.103). No other associations between harboring a *LRRK2* mutation and specific cancers types were uncovered.

**Table 3:** Associations of *LRRK2* mutation with overall cancer outcome and outcome of various cancer types adjusted for age and sex

Cancer outcome	iPD	LRRK2	OR	95% CI	*p*‐value
	*n* (%)	*n* (%)			
Non‐skin	126 (15.1)	18 (17.5)	2.09	1.16–3.77	.015
Colorectal	25 (3.0)	2 (1.9)	1.00	0.23–4.41	.998
Breast – women^a^	11 (3.6)^a^	5 (9.6)^a^	4.58^a^	1.45–14.51	.010
Prostate – men^a^	34 (6.5)^a^	3 (5.9)^a^	1.88^a^	0.52–6.80	.335
Lymphoma/Hematologic	12 (1.4)	2 (1.9)	1.89	0.39–9.18	.430
Meningioma	8 (1.0)	2 (1.9)	2.50	0.48–12.88	.275
Other	18 (2.2)	4 (3.9)	1.95	0.60–6.32	.266

a Percentages and odds ratio (OR) of sex specific cancers are based on the number of men and women.

**Figure 1:**
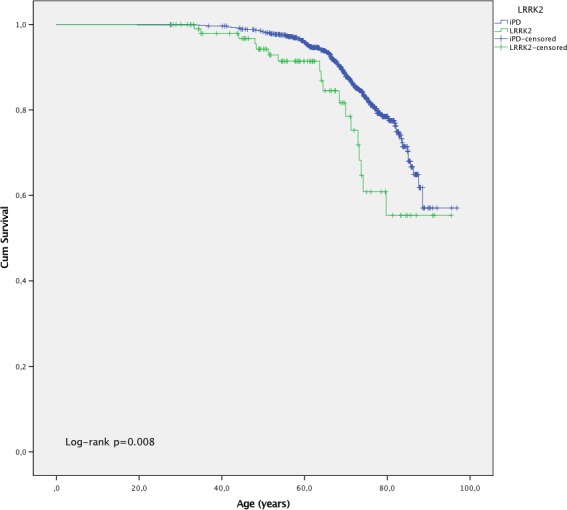
Survival function of all cancer types in *LRRK2* mutation carriers and iPD patients

A Kaplan–Maier survival analysis was conducted to estimate survival curves for age at cancer diagnosis for both *LRRK2* mutation carriers and iPD‐patients (Figure 1). A similar percentage of cases were censored in both groups; 82.5% and 83.5% in the LRRK2 and iPD groups, respectively. The log‐rank test was applied to compare if the survival distributions of age at cancer were different between the two groups. There was a statistically significant difference in survival distribution for the *LRRK2* versus iPD subjects, *p *=* *.008.

## 4 DISCUSSION

*LRRK2* mutation carriers have significantly increased age and sex adjusted risk of non‐skin cancer compared with iPD subjects (OR 2.09; 95% CI 1.16–3.77; *p *=* *.015). This was mainly driven by the increased risk between harboring the mutation and breast cancer in women (OR 4.58; 95% CI 1.44–14.5; *p *=* *.010). No other associations between harboring a *LRRK2* mutation and specific cancer types were uncovered.

*LRRK2* is a large gene of 51 exons. It encodes the multi‐domain cytoplasmic protein termed leucine‐rich repeat kinase 2 (LRRK2) which includes a leucine‐rich repeat toward the N‐terminal (LRR), a kinase domain at the C‐terminal (MAPK), and a Roc (Ras of Complex proteins) domain which encodes a GTPase, and a COR (C‐terminal of Roc) domain (Bernd & Gilsbach, 2014). All are potentially associated with protein–protein interactions (Dachsel & Farrer, 2010; Klein & Westenberger, 2012). The normal function of the LRRK2 protein is still unknown. It is thought that *LRRK2* mutations result in increased kinase activity, which then mediates neuronal toxicity (Smith et al., 2006) although the data regarding kinase activity for PD−related *LRRK2* mutations is conflicting. Increased kinase activity has only been consistently shown for the *G2019S* mutation, while no effect or decreased kinase activity has been reported for the other mutations (Bernd & Gilsbach, 2014). However, the other mutations may increase kinase activity by trapping LRRK2 in a GTP bound active state (Mehdi et al., 2016), and the clear overlap in phenotype between the various mutations indicates a common pathway necessary for pathogenesis.

Kinases are involved in the homeostasis of virtually every cellular process, and are essential regulators of almost every signal transduction cascade (Bernd & Gilsbach, 2014). Perturbation in kinase activity has been implicated in various forms of cancer (Blume‐Jensen & Hunter, 2001). Although the serine/threonine kinases (the family of kinases to which LRRK2 belongs) have received less attention than tyrosine kinases, alterations in the expression of these are likely a relatively frequent occurrence in human tumors (Capra et al., 2006). In addition, LRRK2 amplification has been implicated in renal and thyroid carcinomas (Looyenga et al., 2011). In light of this, the increased risk of non‐skin cancers in *LRRK2* mutation carriers compared with iPD subjects may therefore not come as a surprise. This is also in line with most previous studies (Agalliu et al., 2015; Inzelberg et al., 2012; Saunders‐Pullman et al., 2010). Still the underlying mechanism linking LRRK2 and cancer remains to be elucidated.

Increased risk of breast cancer in PD patients has been reported in several studies. A Danish study based on national hospital discharge and cancer registries, including more than 20 000 PD patients, reported an increased risk of breast cancer in PD patients compared to the general population with a standardized incidence ratio (SIR) of 1.17 (95% CI 1.02–1.34; Rugbjerg et al., 2012). Based on the lower incidence and prevalence of PD in women compared with men (Wirdefeldt, Adami, Cole, Trichopoulos, & Mandel, 2011), association between oophorectomy before menopause and increased risk of parkinsonism (Rocca et al., 2008), reduced risk of PD in estrogen‐treated postmenopausal women (Currie, Harrison, Trugman, Bennett, & Wooten, 2004), and retarded progression of PD in estrogen‐treated postmenopausal PD patients (Marder et al., 1998), a protective role of estrogen in PD has been hypothesized. Epidemiologic and experimental studies indicate that increased concentrations of estrogens are associated with increased risk for breast cancer (Travis & Key, 2003). By continuation, women who develop PD may have been less exposed to estrogens than women who do not develop the condition. Increased risk of breast cancer in female PD patients is therefore puzzling, unless unknown shared risk factors between PD and breast cancer exist. However, the role of estrogens in PD is controversial. Two large prospective cohort studies have recently addressed the possible protective effect of estrogens (Liu et al., 2013; Rugbjerg, Christensen, Tjønneland, & Olsen, 2013). A Danish cohort of almost 27 500 women filled out questionnaires on diet and lifestyle, including reproductive factors, hormone use, and smoking habits. The cohort was then followed up for PD in the Danish Hospital Registry. No significant association between reproductive factors and risk of PD was found. The use of oral contraceptives was associated with non‐significantly increased risk of developing PD (Hazard Ratio [HR] 1.3; 95% CI 0.81–2.09), while the use of hormone replacement therapy was associated with a non‐significantly increased risk of developing PD (HR 1.41; 95% CI 0.90–2.21; Rugbjerg et al., 2013). The results from the Danish study were overall supported by a large US study including almost 120 000 postmenopausal women reporting similar findings (Liu et al., 2013).

The particularly striking result of increased breast cancer risk in LRRK2 versus iPD group in our study corroborates the results reported by Inzelberg et al. (2012) and Agalliu et al. (2015). However, these studies included a skewed distribution of Ashkenazi and non‐Ashkenazi subjects in the *LRRK2* mutation and non‐mutation groups. A possible ethnicity effect could therefore not be ruled out for non‐skin cancer in general and breast cancer in particular. Our study included only ethnic Norwegians, indicating the increased breast cancer risk is independent of ethnicity in the presence of the *LRRK2* mutation.

It has also been attempt to unravel if the association between breast cancer and the *LRRK2* mutation was limited to PD patients. Mortiboys, Cox, Brock and Bandmann (2013) genotyped 1014 breast cancer patients and 1033 controls without PD for the *G2019S* mutation; none were found. The group suggested that *LRRK2 G2019S* does not predispose to breast cancer in the absence of PD in a Western European population. However, the prevalence of the *LRRK2 G2019S* is highly variable in different populations, and very low in the United Kingdom, limiting the validity of this study. Our study included both manifesting and non‐manifesting *LRRK2* mutation carriers. Three of the breast cancer cases were in non‐manifesting carriers, while one of PD patients had breast cancer before PD. In total, 12 LRRK2 PD− had cancer and 4 LRRK2 PD+ had cancer before PD, indicating that cancer in association with the *LRRK2* mutation may not be limited to the presence of PD. However, the disease process underlying PD has probably been ongoing for years before PD is diagnosed, but it is impossible to predict process when the process of phenoconversion starts. It is unknown if manifesting *LRRK2* mutation carriers more or less vulnerable to cancer than never‐manifesting mutation carriers.

A clear advantage of this study was the large, well‐defined, ethnic homogeneous population of 933 individuals with detailed description of demographic and clinical features, and cancer outcomes from the Norwegian Cancer Registry. However, the statistical power to investigate associations between the *LRRK2* mutation and uncommon cancers was limited by the relatively small sample size and small number of some cancers. Lack of information on hormonal and reproductive factors may confound the association between *LRRK2* mutations and breast cancer.

The most important limitation to this study, and the majority of studies assessing the association between harboring a *LRRK2* mutation and developing cancer, is the retrospective screening of cancer outcomes in comparing risk of cancer in iPD versus *LRRK2* mutation carriers. Ideally, a longitudinal study of disease processes should be conducted in which all study participants would be enrolled prior to the first event of interest and followed until the final event was observed. In this way, the entire process is observed for all participants. This is not always feasible as the process of interest may develop over many years, and the age at onset of the process or timing of events may vary considerably for the participants (Cain et al., 2011). This is the case for both cancer and PD, and no biomarker exists to indicate when the pathogenesis begins for either condition. In addition, the *LRRK2* mutation carriers harbor their mutation from birth.

Left truncation is a possible source of bias in our study. Left truncation occurs when an individual who has already passed the event of interest at the time of study recruitment is not included in the study. Only individuals who die before developing PD or becoming known to us because of their *LRRK2* mutation are truly left truncated in our data. We do not know if the truncation frequency is equal among individuals who harbor the *LRRK2* mutation and individuals who are destined to later develop iPD.

Another caveat of this study is including both manifesting and non‐manifesting *LRRK2* mutation carriers, some of whom are related, in one group. *LRRK2* mutations cause autosomal dominant PD, but the penetrance is reduced, age‐dependent, and varies in different ethnic groups (Healy et al., 2008; Hentati et al., 2014). Epigenetic factors, gene–environment interactions, and stochastic events may play a role in who develops PD. These factors may also influence who develops cancer.

Some epidemiologic studies addressing cancer in PD patients have proposed possible underdiagnosis of cancer in PD patients as some cancers require extensive evaluation, which may less likely be performed in a disabled PD patient. This could therefore account for some of the difference between our two groups. On the other hand, PD patients are frequently followed by doctors and may therefore have easier access to cancer evaluation, introducing possible surveillance bias. Hence, the difference between the LRRK2 and iPD groups could be even greater.

The relationship between PD and cancer is complex, but the two apparent opposites may have several mechanisms in common. Further examinations of these mechanisms may provide new insights into and hopefully open for new treatments for both diseases. Assessing the cancer risk in different *LRRK2* mutation types is an interesting path, as the various mutations “affect the formations and activity of the protein LRRK2 in different and specific ways” yet ultimately lead to the same phenotype (Dachsel & Farrer, 2010).

In conclusion, this study confirms increased cancer risk in *LRRK2* mutation carriers compared with iPD subjects. The increased risk is mainly driven by an increased risk of breast cancer in women and is likely independent of ethnicity. The lack of hormonal, reproductive, and environmental data relevant for cancer diagnosis limits the study. Possible bias due to truncation, censoring and shared genetic, environmental and stochastic events in families cannot be ruled out.

## CONFLICTS OF INTEREST

None declared.

## AUTHOR CONTRIBUTIONS

Study design, acquisition of data, statistical analysis, and interpretation of data; drafting and revising the manuscript; and final approval of the manuscript were carried out by BJW. Study conception, design and supervision; acquisition and interpretation of data; revising the manuscript; and final approval by JOA.
